# Extra-axonal contribution to double diffusion encoding-based pore size estimates in the corticospinal tract

**DOI:** 10.1007/s10334-022-01058-8

**Published:** 2023-02-06

**Authors:** Patricia Ulloa, Vincent Methot, Viktor Wottschel, Martin A. Koch

**Affiliations:** 1 Institute of Medical Engineering, University of Luebeck, Ratzeburger Allee 160, 23562 Luebeck, Germany; 2 Department of Radiology and Nuclear Medicine, Amsterdam University Medical Centers, De Boelelaan 1117, 1081, Amsterdam, The Netherlands

**Keywords:** Microstructure, Extracellular space, Size estimates, Multiple wave vector diffusion weighting, Diffusion anisotropy, White matter

## Abstract

**Objective:**

To study the origin of compartment size overestimation in double diffusion encoding MRI (DDE) in vivo experiments in the human corticospinal tract. Here, the extracellular space is hypothesized to be the origin of the DDE signal. By exploiting the DDE sensitivity to pore shape, it could be possible to identify the origin of the measured signal. The signal difference between parallel and perpendicular diffusion gradient orientation can indicate if a compartment is regular or eccentric in shape. As extracellular space can be considered an eccentric compartment, a positive difference would mean a high contribution to the compartment size estimates.

**Materials and methods:**

Computer simulations using MISST and in vivo experiments in eight healthy volunteers were performed. DDE experiments using a double spin-echo preparation with eight perpendicular directions were measured in vivo. The difference between parallel and perpendicular gradient orientations was analyzed using a Wilcoxon signed-rank test and a Mann–Whitney *U* test.

**Results:**

Simulations and MR experiments showed a statistically significant difference between parallel and perpendicular diffusion gradient orientation signals ($\alpha =0.05$).

**Conclusion:**

The results suggest that the DDE-based size estimate may be considerably influenced by the extra-axonal compartment. However, the experimental results are also consistent with purely intra-axonal contributions in combination with a large fiber orientation dispersion.

## Introduction

**Fig. 1:**
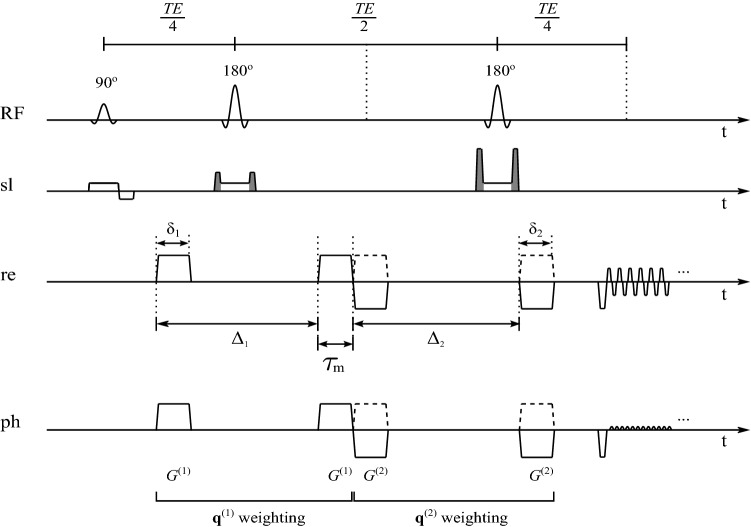
Double diffusion encoding (DDE) imaging sequence with EPI readout (schematic) (sl: slice selection, re: readout, ph: phase encoding). Dotted lines in the RF timeline show the occurrence of echoes. Crusher gradients are shown in the slice selection timeline (in grey), located before and after the refocusing pulses to suppress unwanted coherence pathways. Diffusion gradients drawn with a solid line correspond to an experiment with $\psi =0$ (i.e., $\textbf{q}^{(1)}=\textbf{q}^{(2)}$). Dashed lines show the $\textbf{q}^{(2)}$ weighting in the $\psi =\pi$ case. The pulses are not drawn to scale

Diffusion-weighted magnetic resonance imaging has evolved into a widely used tool for studying tissue microstructure [1–5]. In diffusion-weighted imaging, the magnetization preparation is most often based on the pulsed gradient spin echo [6]. In recent years, modifications of this approach have been proposed. These usually aim at making the experiment sensitive to various aspects of tissue microstructure, exploiting the unique sensitivity of diffusion-weighted magnetic resonance to the geometry of obstacles to molecular diffusion, such as cell membranes. The terms double diffusion encoding (DDE) [7] and double wave-vector (DWV) diffusion weighting [8] are used for a straightforward extension of the conventional Stejskal–Tanner [6] diffusion-weighting. Here, two (instead of one) pairs of diffusion-sensitizing gradient pulses are applied between excitation and acquisition (see Fig. 1) [8–10]. Apart from the conventional acquisition parameters available for both SDE and DDE, such as gradient duration ($\delta _1$ and $\delta _2$) and strength ($G^{(1)}$ and $G^{(2)}$), and diffusion time ($\Delta _1 - \delta _1/3$ and $\Delta _2 - \delta _2/3$), the DDE set-up provides two additional degrees of freedom: it is possible to vary the mixing time, $\tau _\text {m}$, separating the two diffusion-weighting periods, and the angle, $\psi$, between the two corresponding gradient directions. This may permit to study microscopic tissue characteristics that are not easily accessible using other non-invasive methods. DDE experiments are sensitive to different aspects of tissue microstructure, such as microscopic anisotropy [11, 12], perfusion fraction [13], pore size [14], and molecular exchange [15, 16]. However, it is difficult to design an experiment providing a measure that is specific to a single effect out of these. Experiments that compare between the effects of Stejskal-Tanner weighting and more complex gradient waveforms were proposed to assess the shape of microscopic compartments [17, 18]. Previous DDE experiments to estimate the size of compartments (e.g., cells) using the signal difference between parallel and antiparallel diffusion gradients have been performed in the water-filled spaces around polymer beads, in radish root, and fixed porcine spinal cord [19–21], in microcapillaries [21–24], fixed rat spinal cord [25], plant cells [26], yeast cells [27], and in human brain in vivo [28–30].

Early applications of DDE weighting for an assessment of compartment size in vivo seemed to result in relatively large diameters [28], although some degree of overestimation of axon diameters can be expected on theoretical grounds [31]. Preliminary work on patients with stroke-induced Wallerian degeneration of neurons in the corticospinal tract (CST) revealed a reduced signal difference between parallel and antiparallel gradient orientations in the affected tract [32]. These in vivo results on human brain tissue raised the question to which degree the extracellular space contributes to the compartment size estimates gained by evaluating the DDE signal.

On the other hand, it was suggested that DDE experiments may provide further information about pore shape when using long mixing times [8, 9, 14, 33–35]. This approach was used in recent years to detect “microscopic anisotropy” in tissue which macroscopically appears isotropic in conventional (i.e., single diffusion encoding) diffusion-weighted measurements [29, 36–38].

Clinical applications of this approach were also investigated [39]. Considering double diffusion encoding experiments on a sample comprising eccentric but randomly oriented water-filled pores, Mitra [8] distinguished between two effects. The “filter effect”, leading to an attenuation difference between parallel and perpendicular gradient orientations, which is exploited in studies assessing microscopic anisotropy, is independent of the mixing time. In contrast, the difference between parallel and antiparallel gradient orientations, which is exploited in attempts determining the pore or cell size, depends on $\tau _{\text {m}}$ being short. It should be noted that single and double diffusion encoding experiments in principle yield equivalent information at low gradient amplitude, i.e., when only terms up to second order in the time integral of a single gradient pulse are considered [40].

The goal of this work is to investigate a possible explanation for the overestimation of compartment size in CST in vivo. We hypothesize that the extracellular space contributes considerably to this overestimation. However, correcting for this influence is outside the scope of the present work. In this work, rather the sensitivity of DDE experiments to pore shape is used to acquire information on the relative contribution of the extracellular space to the DDE-derived size estimates in applications to brain white matter, specifically in the CST.

Some of the in vivo results were published previously [41]. In this work, we extend the theory and analysis presented in [41] by allowing for fibers of different inclination in an imaging voxel. We complement the in vivo results shown in [41] (1 volunteer added) with computer simulations based on diffusion tensor imaging (DTI) data acquired for the additional volunteer. Additionally, left-right CST asymmetries are investigated in the present paper.

**Fig. 2:**
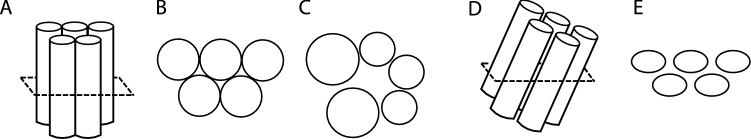
**A** Packed circular cylinders, **B** cross-section of the situation in (**A**), **C** possible cross-section with looser packing, **D** tilted cylinders with circular base, and **E** cross-section of (**D**) (exaggerated), or of untilted cylinders with elliptical base. The signal may depend differently on experimental parameters if water is only present inside the cylinders or both inside and outside. Figure partially adapted from [41]

**Fig. 3:**
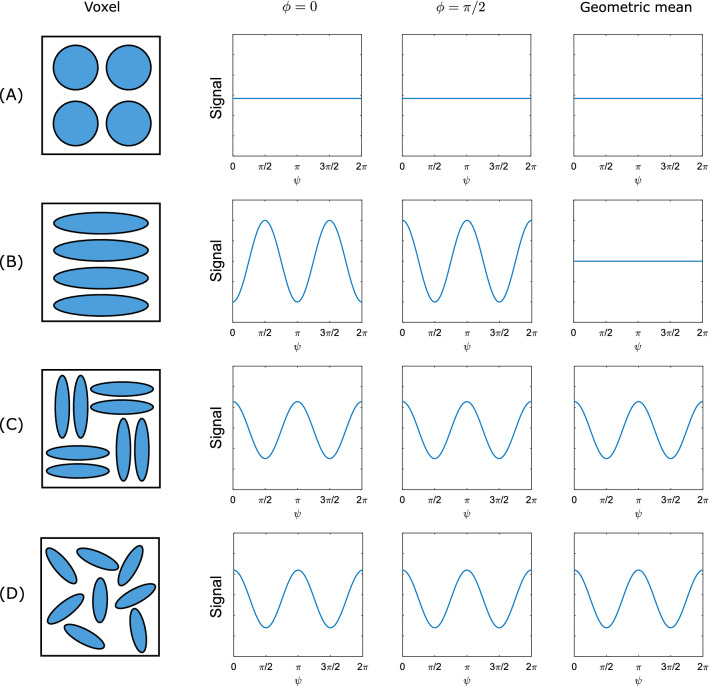
Schematic illustration of the expected angular signal dependence for a DDE sequence at long mixing time, $\tau _{\text {m}}$, for a voxel containing water-filled pores of different shapes. $\psi$ is the angle between the two diffusion gradient wave vectors, $\textbf{q}^{(1)}$ and $\textbf{q}^{(2)}$. $\phi$ is the angle between $\textbf{q}^{(1)}$ and the horizontal axis in the schematic of the voxel microstructure (left). **A** For spherical compartments, no angular dependence is expected. **B** For aligned ellipsoids, the DDE signal modulation depends on $\phi$ and $\psi$, exhibiting a $\cos (2\psi )$ angular dependence. However, for **(C)** and **(D)** (aligned ellipsoids perpendicular to each other and randomly oriented ellipsoids, respectively), the DDE signal will show a $\cos (2\psi )$ dependence, and it does not depend on $\phi$. The column on the right shows the geometric mean of the $\phi =0$ and $\phi =\pi /2$ columns. The geometric mean cancels out the parallel–perpendicular difference in (**B**) but not in (**C**) and (**D**). The plots would show the same qualitative behaviour if the voxel contained cylindrical pores instead, with the schematic on the left showing the cross section of the pores

## Theory

*Basic theory*: DDE experiments consist of two pairs of diffusion-sensitizing gradients separated by a mixing time, $\tau _{\text {m}}$ (see Fig. 1). Mitra [8] developed the first theoretical description of DDE. He described two $\tau _{\text {m}}$ time regimes, $\tau _{\text {m}} = 0$ and $\tau _{\text {m}} \rightarrow \infty$, under idealized parameter timing conditions ($\delta \ll \tau _D \ll \Delta$, where $\tau _D = (2a)^2/(2D_0$) is the mean time for a particle to move across a compartment of diameter 2*a*, and $D_0$ is the free diffusion coefficient). For $\tau _{\text {m}}=0$, a cosine-shaped signal modulation is expected for restricted compartments independent of their shape. The modulation amplitude (specifically, the parallel–antiparallel signal difference) is proportional to the square of the pore size. This is because at short $\tau _{\text {m}}$ ($\tau _{\text {m}}\ll \tau _D$), the spins paths are not completely independent of each other: the particle positions at the end of the first and the start of the second weighting are almost the same. Hence, the re- and dephasing gradient pulses of the two weighting periods can partly cancel or reinforce each other [8, 42]. However, for long $\tau _{\text {m}}$ ($\tau _{\text {m}}\gg \tau _D$) this correlation between the spin paths in successive weighting periods is lost. Then, a $\cos (2\psi )$ signal modulation due to eccentric pores will still be observable and no signal modulation is left for spherical pores. At short $\tau _{\text {m}}$, the $\cos (\psi )$ and $\cos (2\psi )$ modulations are superimposed, with individual amplitudes.

The experiments presented are designed to use a low $\tau _{\text {m}}$ value, $\tau_{\text{m}}=\delta +t_{\text{r}}$, with $t_{\text {r}}$ being the gradient ramp time. (Shorter values would mean overlapping gradient pulses.) This has the advantage that it is possible to use the signal modulation for size estimates (parallel–antiparallel difference, still observable at low $\tau _{\text {m}}$) and for microscopic anisotropy (parallel-perpendicular difference, independent of $\tau _{\text {m}}$).

(1) *Objective and problem description* In this article, we are concerned with size estimates which exploit the DDE signal difference between parallel and antiparallel gradient orientations at short $\tau _\text {m}$ values (size effect). Previous published results employing this approach in the human CST resulted in an overestimation of the axonal diameter [28].

Here, we attempt to find out whether the intra- or extra-axonal compartment is the main signal source in those experiments, by using the parallel–perpendicular DDE signal difference at intermediate $\tau _\text {m}$. Figure 3 depicts schematically the expected angular dependency for four generalized voxel scenarios at long $\tau _{\text {m}}$. If the origin of the signal is eccentric, as expected for the extracellular space, we will observe a $\cos (2\psi )$ signal modulation (shape effect) as shown in Fig. 3C). On the other hand, if this signal modulation is not present, then we can assume that the origin of the signal arises from a non-eccentric compartment, as seen in Fig. 3A, consistent with the shape of the intracellular space. Nevertheless, in the CST, this is not that simple, because in the plane spanned by the diffusion gradients, intra- and extracellular space appear as eccentric compartments [43]. We explain how the signal's geometric mean between diffusion gradients rotated by $\pi /2$ can help us to identify the origin of the MR signal. In the following, we assume long $\tau _\text {m}$.

(2) *Mitra’s condition (i), long *$\tau _{\text {m}}$ In principle, DDE measurements in the long $\tau _{\text {m}}$ regime can be used to detect whether water in a sample is confined to spherical or eccentric compartments [9]. At long $\tau _{\text {m}}$, water-filled spheres yield the same DDE signal for parallel and perpendicular gradient orientations. In contrast, eccentric pore shapes, such as ellipsoids, exhibit different signal amplitude in cases of parallel and perpendicular gradient orientations, even if the pores are randomly oriented (see Fig. 3) [8]. Hence, DDE experiments can be used to gather information on the pore shape.

(3) *Theoretical assumptions* In coherent white matter, let us assume that the diffusion gradients are applied in a plane perpendicular to the axonal axis. For our purposes, the white matter can be seen as a bundle of densely packed, infinitely long cylinders with circular base, as depicted in Fig. 2A). A perpendicular cross-section through such a bundle of same-size cylinders consists of circles. The compartments between these circles resemble equilateral triangles—this shape is sometimes called circular horn triangle. Unfortunately, as the circular horn triangle possesses a 3-fold symmetry axis (see Fig. 2A, B), it is not expected to induce a measurable signal difference between parallel and perpendicular gradient orientations in DDE experiments. Such pores cannot easily be distinguished from circular compartments, i.e., pores with $C_\infty$ symmetry. (This might be possible, however, using approaches designed to image the mean pore shape directly [44].) Nonetheless, as presented in Ulloa et al. [41], if the cylinders are different in size and not too densely packed, the cross-section of the space between the cylinders is “irregularly shaped” (Fig. 2C), meaning here that no rotational symmetry exists. If sufficiently large, the deviation from a circle can be detected by DDE experiments with long $\tau _{\text {m}}$, comparing the signal for parallel and perpendicular diffusion gradient directions, with the diffusion gradients oriented in a plane perpendicular to the fiber axis. This is expected to work even in case of open pores in the plane spanned by the diffusion gradients [8]. Pores with circular cross-section, on the other hand, will not exhibit a parallel–perpendicular signal difference in such a DDE experiment. Hence, the parallel–perpendicular signal difference might yield information about the shape of the compartment dominating the DDE pore size estimate in the CST.

(4) *Reasoning behind the difference between parallel and perpendicular DDE signal* Consider a sample or voxel containing two sets of the same number of equally shaped, eccentric pores. The pores in each set may be all aligned along a given direction, the alignment directions of the two sets being perpendicular to each other. The signal attenuation may be written as $E(q,\psi )=S(q,\psi )/S_{0}$, where $S_0$ represents the signal with all diffusion gradients set to zero, *q* is the identical amplitude of both wave vectors, $\textbf{q}^{(i)}=\gamma \delta \textbf{G}^{(i)*}$, $i=1, 2$, where $\textbf{G}^{(i)*}$ denotes the field gradient vector associated with the first pulse in the effective diffusion-weighting gradient waveform of the $i^{\text {th}}$ weighting, and $\psi$ is the angle between the two $\textbf{q}$ vectors [7]. For parallel ($\parallel$) and perpendicular ($\perp$) orientations of the diffusion wave vector, the attenuations may be expressed employing effective diffusion coefficients,

1 $$\begin{aligned} E_{\parallel } = E(q,0)= \frac{1}{2} e^{-bD_{1}}e^{-bD_{1}} +\frac{1}{2} e^{-bD_{2}}e^{-bD_{2}}, \end{aligned}$$

and

2 $$\begin{aligned} E_{\perp } = E(q,\pi /2)= \frac{1}{2} e^{-bD_{1}}e^{-bD_{2}} +\frac{1}{2} e^{-bD_{2}}e^{-bD_{1}}, \end{aligned}$$

respectively, which in general are not equal, as shown in a more general form by Callaghan and Komlosh (Eq. 3 in ref. [33]). In these equations, $D_{1}$ and $D_{2}$ are the effective diffusion coefficients due to restriction along the two perpendicular diffusion gradient directions involved. The degree of diffusion weighting, $b=\gamma ^{2}G^{2}\delta ^{2} (\Delta -\delta /3)$, depends on the amplitude, *G*, and duration, $\delta$, of the gradient pulses and on their temporal separation in each weighting period, $\Delta$ (see Fig. 1). $E_\Vert$ and $E_\perp$ are also unequal in general if the eccentric pores in the sample are not aligned along perpendicular directions but rather randomly oriented. The inequality reflects the physical basis that makes eccentricity estimation in DDE experiments possible [9, 33].

(5)* In vivo situation in CST* In a fiber bundle in brain white matter, the complication arises that the plane spanned by the diffusion gradients may not be perfectly perpendicular to the fibers. In this case, the cross-section of a circular cylinder is elliptical (Fig. 2D, E). This means that the DDE signal can exhibit a parallel–perpendicular difference even if the signal purely arises from the interior of the circular-base cylinders. Such an observation could be erroneously interpreted as an indication that the signal arises from the irregularly shaped exterior compartment. Hence, we search for means to remove the parallel–perpendicular DDE signal difference if it is simply due to the cylinders not being perpendicular to the diffusion gradient plane, while retaining the difference if it arises from the irregularly shaped exterior compartment.

(5a) *Removing the influence of tilted fibers* To this aim, we consider the 2D cross-sections of pores in the plane spanned by the diffusion gradients, as shown in Fig. 3. In a scenario where a voxel contains only a single orientation of eccentric pores (see Fig. 3B),

3 $$\begin{aligned} E_{\parallel }=e^{-bD_{1}}e^{-bD_{1}} \ne e^{-bD_{1}}e^{-bD_{2}} = E_{\perp } \end{aligned}$$

can occur. The eccentric 2D pore shapes can arise from cylinders with an identical elliptical base, with the cylinder axis perpendicular to the plane, or to cylinders with a circular base that are not perpendicular to the plane. Rotating all gradients by $\pi /2$ will then yield

4 $$\begin{aligned} E'_{\parallel }=e^{-bD_{2}}e^{-bD_{2}} \ne e^{-bD_{2}}e^{-bD_{1}} = E'_{\perp }. \end{aligned}$$

Here and in the following, a primed quantity $A'$ represents the same as *A* but with all gradients rotated by $\pi /2$ in the plane of the diffusion gradients. When taking the geometric mean of each side of Eqs.3) and (4), the signal difference between parallel and perpendicular gradient orientations should vanish, i.e.

5 $$\begin{aligned} \sqrt{E_{\parallel }E'_{\parallel }}=\sqrt{E_{\perp }E'_{\perp }}. \end{aligned}$$

If the difference remains, i.e., if Eq. (5) does not hold, then it can be inferred that the signal does not arise from parallel circular cylinders as depicted in Fig. 2D. The sample may then rather comprise parallel cylinders with eccentric bases that are not aligned with each other (see Fig. 3C), or circular cylinders with more than one direction of inclination (see Fig. 3D). In tissue exclusively comprising parallel cylindrical pores (of arbitrary base), it could then be concluded that the space dominating the signal is irregularly shaped (i.e., is not a cylinder with circular base), such as the extra-axonal space in coherent white matter. This would point to the extracellular space as the origin of the DDE signal. However, it is known that considerable fiber orientation dispersion exists in the corticospinal tracts, even in inferior parts [45]. This means that a parallel–perpendicular DDE difference between signals geometrically averaged over two gradient orientations with a relative $\pi /2$ rotation, can also be caused simply by fiber orientation dispersion (see Appendix A). Unfortunately, a firm conclusion regarding the origin of the signal difference will then not be possible, and further investigation will be required. This is a major drawback of the approach presented here (see Discussion). If, in contrast, no parallel–perpendicular attenuation difference were found after taking the geometric mean, this would point to a purely intra-axonal origin.

It should be noted that the rotationally invariant indices [12, 38] proposed for assessing pore shape cannot be used without modification for the question investigated here: it is beyond question that the compartments (probably both intra- and extracellular space) in white matter are not spherical. The question addressed by DDE means is rather whether the cross-section of the compartments is circular or eccentric, in order to determine whether the DDE size estimate rather refers to the intra- or extra-axonal space. However, the approach used here is based on the idea to average out the influence of macroscopic anisotropy, which is also used in different microscopic anisotropy indices suggested [12, 38].

(5b) *Exceptions* This simple reasoning must be changed if pores of different shapes are present. This would be the situation in a white matter voxel containing loosely packed axons and the plane of the diffusion gradients is not perpendicular to the axonal fibers, as depicted in Fig. 2D, E. The geometric mean approach cannot suppress the signal modulation due to cylinder inclination if the voxel contains cylinders inclined in different directions. This complication was not mentioned in [41] , and it is considered in more detail in Appendix A.

(6) *Summary* In a voxel containing two kinds of pore shapes (eccentric and non-eccentric), the difference between parallel and perpendicular gradient orientations after taking the geometric mean of the DDE signals with all diffusion gradients rotated by $\pi /2$ (in the laboratory frame) will persist in two cases: apart from aligned eccentric compartments, there is a significant contribution of (i) non-eccentric pores or (ii) of unaligned eccentric compartments. In the following, we use a “(g)” superscript to denote the geometric mean, as in

6 $$\begin{aligned} \bar{E}^{(g)}_\Vert =\sqrt{\bar{E}_\Vert \bar{E}'_\Vert } \quad \text {and}\quad \bar{E}^{(g)}_\perp =\sqrt{\bar{E}_\perp \bar{E}'_\perp }, \end{aligned}$$

where $\bar{E}$ corresponds to the arithmetic mean of the DDE attenuations, *E*, for parallel and antiparallel gradient orientations. This arithmetic mean reduces the influence of the size effect. This is explained in detail in Appendix A.

## Methods

### Double diffusion encoding sequence

DDE measurements were performed as described in [41]: using a diffusion-weighted double-spin echo magnetization preparation (see Fig. 1) with EPI read-out on a whole-body MRI system operating at 3 T magnetic field strength (Ingenia 3.0T, Philips, Best, The Netherlands), using an 8-channel head coil array. SPIR fat suppression [46] was employed. All RF pulses were slice-selective. All crusher gradient pulses had the same duration and were applied in the slice-select direction, before and after the refocusing RF pulses. The amplitudes of the crusher pulse pairs differ by a factor of two to suppress unwanted coherence pathways.

In all DDE experiments, the diffusion gradients were always perpendicular to the slice-select direction (in the *x*–*y* plane). This approach avoids cross-terms [47] with the crusher and slice-selection gradients (applied in the *z* direction). Both the angle $\psi$ (angle between the wave vectors), $\textbf{q}^{(1)}$ and $\textbf{q}^{(2)}$, and the angle $\phi$, determining the orientation of $\textbf{G}^{(1)}$ in the laboratory frame, were varied. $\phi =0$ was chosen to correspond to $G_x^{(1)}=G_y^{(1)}$ and $G_z^{(1)}=0$. Mixing times below $\tau _{\text {m}}=\delta +t_{\text {r}}$, with $t_{\text {r}}$ being the gradient ramp time, were avoided in all experiments, in order to exclude overlap between trapezoidal gradient pulses.

### Simulations

Simulations were performed using the software package MISST [48–51] (version v0.93) with Matlab2015b (The MathWorks, Natick, Massachusetts, United States) to study the effect of the relative volume fractions of extra- and intracellular compartments on the DDE signal.

MISST is a semi-analytical simulation tool based on the matrix formalism approach developed by Callaghan [52]. The experimental parameters were set to values that are achievable on a clinical MR system, matching the settings in the in vivo experiments ($\delta =10$ ms, $\Delta =62$ ms, $b=2\cdot 812~\text{s}\,\text{mm}^{-2}$, and $\tau _\text {m}=10.9$ ms).

**Fig. 4:**
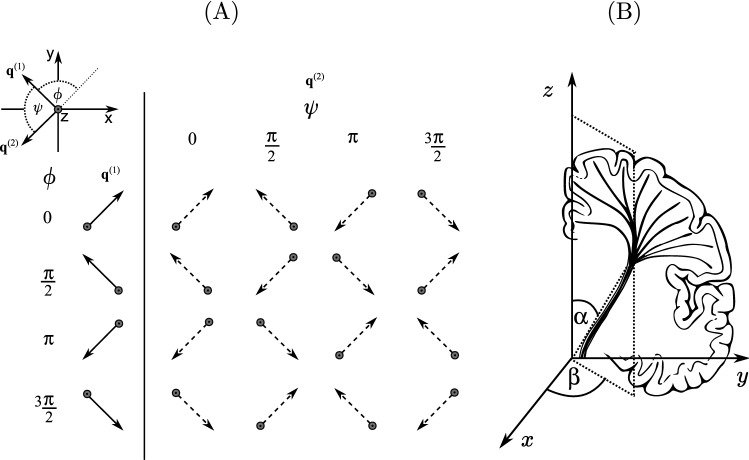
**A** Overview of the diffusion gradient directions in the *x*-*y* plane used in the in vivo experiments. Sixteen different combinations of diffusion gradient orientations were used, where for a given angle $\phi$, **q**$^{(1)}$ (solid line) is fixed and **q**$^{(2)}$ (dashed line) is rotated about an angle $\psi$. **B** Schematic of the DTI-derived angles. $\alpha$ corresponds to the inclination angle of the CST axis with respect to the *z*-axis, and $\beta$ specifies how the projection of the CST axis is oriented in the *x*–*y* plane

A two-compartment model was used to represent the white matter structure in the CST, where the total MR signal originates from water inside the axons or in the extracellular space. The intracellular space was modeled as circular cylinders of diameters 1, 5, and 10 $\mu$m which are inclined with respect to the *z*-axis. Diffusion tensor imaging was performed in vivo (see below) to obtain the inclination angle, $\alpha$, of the axons in the CST (mean of angle modulus over bilateral CST ROI), together with the azimuthal angle, $\beta$, of the axonal axis’ projection onto the *x*–*y* plane, as depicted in Fig. 4B. The DTI-derived angles ($\alpha$ and $\beta$) are used in the simulations. The experimental parameters of the DTI acquisition are described in the next section, “In vivo experiments”. The intrinsic diffusivity inside the cylinders was set to the free self-diffusion coefficient [53] of bulk water at $36\,^{\circ }$C, $D_0=3\cdot 10^{-9}~\text {m}^{2}\,\text {s}^{-1}$.

Diffusion in the extracellular space was described by an effective diffusion tensor. The diffusion tensor has six degrees of freedom: one parallel and two perpendicular diffusivities and three angles ($\alpha$, $\beta$, and $\zeta$) describing the orientation of the tensor. The parallel diffusivity ($\lambda _\text {par}$, the largest tensor eigenvalue) and perpendicular diffusivities ($\lambda _\text {per1}$ and $\lambda _\text {per2}$) were obtained from the DTI data in the bilateral ROI for volunteer no. 8: $\lambda _\text {par}=1.35\cdot 10^{-9}~\text {m}^{2}\,\text {s}^{-1}$, $\lambda _\text {per1}=0.57\cdot 10^{-9}~\text {m}^{2}\,\text {s}^{-1}$, and $\lambda _\text {per2}=0.34\cdot 10^{-9}~\text {m}^{2}\,\text {s}^{-1}$. The largest eigenvalue eigenvector of the tensor was aligned with the symmetry axis of the cylindrical intracellular compartment, given by the DTI-derived angles $\alpha$ and $\beta$. The simulation requires a third angle, $\zeta$, which describes the rotation of the tensor about its axis. Here, it was set to 0. Simulations were performed with $\phi =0$ and $\phi =\pi /2$ and the $\psi$ values used in vivo. The simulated DDE signals for $\phi =0, \pi /2$ were averaged using a geometric mean. The simulations were run with three intracellular volume fractions, $f_i=1$ (only cylinder), 0.7, 0.5, 0.3 and 0 (only tensor). Compartment sizes were estimated from the simulated data using Eqs. 10) and (11). Simulations with varying $\tau _{\text {m}}$ (with fixed cylinder diameter $d = 10~\mu$m) were also performed in order to show the signal dependence on $\tau _{\text {m}}$. The result is given in Appendix C. Additionally, simulations using ideal sequence parameters are presented in Appendix D.

### In vivo experiments

DDE experiments were performed on eight healthy volunteers (four female, four male; mean age $25.75\pm 2.31$ years, all right-handed) without known history of neurological disease. Written informed consent was given before data acquisition. Sixteen choices of the diffusion gradient directions were used as depicted in Fig. 4A. For data analysis, a ROI was used consisting of two parts, comprising the left and right corticospinal tracts in the slices used for diffusion imaging. The ROI was obtained by applying a threshold to the average of all diffusion-weighted images. The threshold was set at 50 % of the difference between the white matter signal outside of and within the CST. Voxels that obviously did not belong to the CST were manually removed. Twenty axial slices of 3 mm thickness were acquired using $3 \times 3~\text {mm}^{2}$ nominal in-plane resolution. Echo and repetition times were $T\!E=180$ ms and $T\!R=6.5$ s. An additional experiment was performed with volunteer no. 7 ($T\!E=200$ ms, $T\!R=6.5$ s), using a longer $T\!E$ in order to accommodate a longer mixing time $\tau _{\text {m}}=25.9$ ms, in order to compare this to the experiment with the shorter $\tau _{\text {m}}=\delta +t_{\text {r}}= 10.9$ ms.

The acquisition parameters were $\delta =10$ ms, $\Vert {\textbf {G}}\Vert =G^{(1)}=G^{(2)}= 44~\text {mT\,m}^{-1}$, $\Delta =62$ ms (satisfying $\Delta \gg \tau _D$), gradient rise time $t_{r}=900~\mu$s, $\tau _\text {m}=\delta +t_\text {r}$ for all eight volunteers, corresponding to a total diffusion weighting of $b=2\cdot 812~\text {s\,mm}^{-2}$ (and 0), and 15 repetitions. These parameters were chosen as close as possible to the in vivo measurements in Koch et al. [28], which violates the conditions assumed by Mitra [8] ($\delta \ll \tau _D$ and $\tau _{\text {m}}=0$). However, numerical simulations have shown that the parallel–antiparallel signal difference is still observable for micrometer-size pores [54]. The mixing time, $\tau _\text {m}$, corresponds to the minimal duration possible with our experimental parameters and hardware. The chosen $\tau _\text {m}$ allowed us to observe parallel–antiparallel and parallel–perpendicular signal differences, for assessing size and compartment shape in a single experiment.

In addition, anatomical $T_1$-weighted images were acquired using turbo field echo (TFE), and DTI was employed for estimating the fiber orientation in the tissue, to be used in the computer simulations using MISST. In the DTI acquisition, a diffusion-weighted spin echo preparation with EPI read-out was employed, using $b=0$ and $800~\text{s}\,\text{mm}^{-2}$ with 32 gradient directions. SPIR fat suppression [46] was also used here. The effective echo time was $T\!E=60$ ms, $T\!R=14.6$ s, FOV $224\times 224~\text {mm}^{2}$, voxel size $2.1\times 2.1~\text {mm}^{2}$, 70 axial slices with a thickness of 2 mm. The scan time required for anatomical T1-weighted images and DTI measurements was approximately 5 and 10 min, respectively. The acquisition time for each DDE experiment was approximately 30 min. Usually, the total acquisition time was close to one hour. However, sometimes volunteer repositioning was needed or further protocol optimization. Then, we made sure that the total measurement time did not exceed 2 h.

### Data analysis

The in vivo images were realigned to the non-diffusion weighted image to correct for subject motion, using the Diffusion toolbox for SPM12 in Matlab R2015b (The MathWorks, Natick, Massachusetts, United States).

For DTI and DDE, the realignment in the in vivo images was based on the non-diffusion weighted images. Any motion between acquiring the $b=0$ images was not taken into consideration. In DTI only, the effect of motion correction on the value of the diffusion gradient components was accounted for [55]. For $\tau _{\text {m}}=0$ and small *q*, the diffusion signal can be approximated by [8]

7 $$\begin{aligned} E(q,\psi )= & {} \frac{S(q,\psi )}{S_0} = \nonumber \\{} & {} 1 - \frac{1}{3} R_\text {g}^2 q^2 (2-\cos (\psi )) + {\mathcal {O}}(q^4), \end{aligned}$$

where $R_\text {g}^2$ is the mean-squared radius of gyration, scaling with pore size, and $q=\gamma \delta G$. Note the sign in front of $\cos (\psi )$ differs from the original publication [8] since in the present article the conventions in [7] are used. Calculating the signal attenuation using Eq. (7) for the parallel ($\psi =0$) and antiparallel ($\psi =\pi$) cases results in

8 $$\begin{aligned} E(q,0) = 1 - \frac{1}{3} R^2_g q^2 \quad \text {and}\quad E(q,\pi ) = 1 - R^2_g q^2 \end{aligned}$$

(${\mathcal {O}}(q^4)$ terms omitted). Then, an estimate of the pore size can be calculated from the parallel–antiparallel attenuation difference [19, 28],

9 $$\begin{aligned} R_g^2 = \frac{3}{2} \frac{E(q,0)-E(q,\pi )}{(\gamma \delta G)^{2}}. \end{aligned}$$

In order to reduce the effect of macroscopic anisotropy, the geometric mean of two measurements with all gradients rotated by $\pi /2$ was used in the present study to calculate

10 $$\begin{aligned} R_\text {g}^2 = \frac{3}{2} \frac{(E(q,0)E'(q,0))^{1/2}-(E(q,\pi )E'(q,\pi ))^{1/2}}{(\gamma \delta G)^{2}}. \end{aligned}$$

In the limiting case with $\delta \ll \tau _D\ll \Delta$, $\tau _\text {m}=0$, and $(qa)^2\ll 1$, where “*a*” is a typical pore size and $\tau _D$ is the mean time required for diffusion across the pore, the quantity $R_\text {g}^2$ yields the mean squared radius of gyration if the sample consists of randomly oriented closed pores [8]. With the assumption that the pores are identical upright cylinders with circular base, it can be translated to a diameter estimate,

11 $$\begin{aligned} 2r_\text {lim}=2\sqrt{R_\text {g}^2 \frac{2}{3} c}, \end{aligned}$$

with $c\approx 2$. This can be found by using the analytic signal expression for untilted cylinders [56, Eq. (15)] for the attenuations, *E*, in Eq. (9) with $\tau _{\text {m}}=\delta$ and $\Delta \rightarrow \infty$, retaining the first term of the infinite series only, and approximating $\exp (-\omega _{2,1}\delta )\approx 1-\omega _{2,1}\delta$. By using the geometric mean over gradient orientations that are rotated by $\pi /2$ with respect to each other, the dependence of the attenuation on the fiber direction is removed (if the axons can be represented as cylinders). This can be inferred from the analytic expressions for the attenuation in infinite cylinders accounting for an inclination towards the diffusion gradients [56]. When comparing the in vivo results with previous results in Koch and Finsterbusch [28], it should be noted that in the latter study an arithmetic average over the DDE signals with the diffusion gradients rotated by $\phi =\pi /2$ was employed.

The difference between parallel and perpendicular gradient orientations after taking the geometric mean over gradient settings rotated by $\pi /2$ is given by

12 $$\begin{aligned} \begin{aligned}&\bar{E}^{(g)}(q,0)- \bar{E}^{(g)}(q,\frac{\pi }{2}) &= \left[ \frac{(E(q,0)+E(q,\pi ))(E'(q,0)+E'(q,\pi ))}{4}\right] ^{\frac{1}{2}}- \\&\left[ \frac{(E(q,\frac{\pi }{2})+E(q,\frac{3\pi }{2}))(E'(q,\frac{\pi }{2})+E'(q,\frac{3\pi }{2}))}{4}\right] ^{\frac{1}{2}}. \end{aligned} \end{aligned}$$

For each volunteer, four hypotheses about the individual voxel data were statistically tested: (T1) $H_{1,1}:\bar{E}^{(g)}(q,0)>\bar{E}^{(g)}(q,\pi /2)$ in a region of interest of the image comprising the right CST; (T2) as in T1 but for the left CST; (T3) as in T1 but for a region of interest comprising the bilateral corticospinal tracts; (T4) $H_{1,4}:R_\text {g}^2$ is larger in the right than in the left corticospinal tract ROI. For the tests T1 to T3, a Wilcoxon signed-rank test was applied, while in T4, a Mann Whitney U test was used. For all statistical tests, the significance level $\alpha =0.05$ was chosen.

## Results

### Simulations

**Fig. 5:**
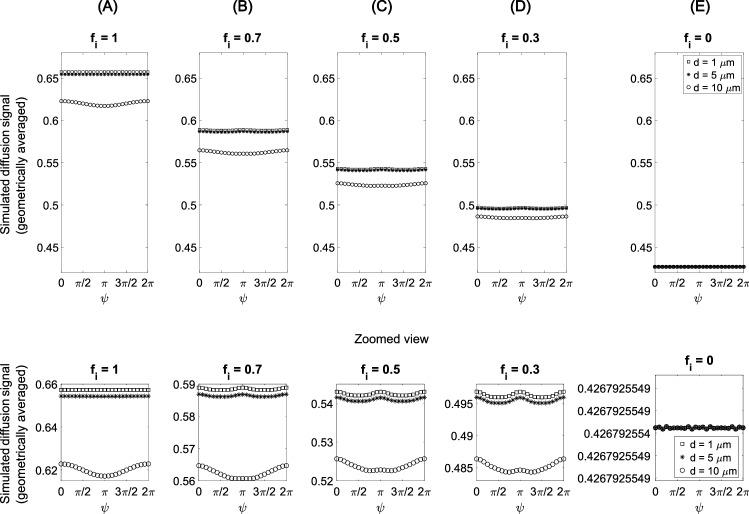
Simulated diffusion signal of different volume fractions after taking the geometric mean over the $\phi =0$ and $\phi =\pi /2$ cases. The intracellular space (volume fraction $f_{i}$) is represented by cylinders of three different diameters, and the extracellular space is described by a diffusion tensor (diffusivities obtained from the DTI analysis performed on volunteer no. 8). **A** $f_{i}=1$ (no extracellular component), **B** $f_{i}=0.7$, **C** $f_{i}=0.5$, **D** $f_{i}=0.3$, and **E** $f_{i}=0$ (only extra-axonal component). The second row shows a zoomed view of the plots in the first row. For considerable intracellular volume fractions (columns **B**, **C**, **D**), the simulations show a slight W-shaped modulation, in particular for small cylinder diameters. The W-shaped modulation arises from the mixed signal of cylinder and tensor

Simulations using MISST were performed to study the DDE diffusion signal in idealized compartments and to compare them with the experimental MR results. The simulation results are shown in Fig. 5. The measured DTI data was used to extract the angle of inclination, $\alpha$, of the CST main axis with respect to the *z*-axis, and the azimuthal angle, $\beta$, of its projection on the *x*-*y* plane (measured from the *x*-axis, see Fig. 4B). The simulations were based on the angles obtained from the DTI data for volunteer no. 8 ($\alpha =25^{\circ }$ and $\beta =45^{\circ }$, using the diffusion tensor’s eigenvector corresponding to the largest eigenvalue as an estimate for the CST axis orientation. The mean value over all volunteers was $(24.68 \pm 2.38)^{\circ }$ for $\alpha$ and $(48.73 \pm 2.48)^{\circ }$ for $\beta$ (± standard deviation between volunteers).

To study the signal behavior for different intra-axonal volume fractions, the simulated signals from tilted cylinders and from tensor-described compartments were summed up. The results are shown in Fig. 5. In the first plot, corresponding to a situation without extracellular space, it can be seen that after the geometric mean over perpendicular orientations in the laboratory frame (specified by $\phi$), the signal behaves just as if it came from untilted cylinders, showing a characteristic $\cos (\psi )$ profile if the cylinder diameter is large enough. This is because the parallel–antiparallel DDE signal difference is proportional to $R^2_g$, scaling with compartment size. For higher extracellular volume fractions, a W-shaped profile, $\cos (2\psi )$, occurs for cylinders with diameters below 10 $\mu$m.

**Table 1:** Size estimates calculated from simulated data

	$2r_{\text {lim}}$ / $\mu$m				
True compartment size	$f_i=1$	$f_i=0.7$	$f_i=0.5$	$f_i=0.3$	$f_i=0$
$d = 1\,\mu$m	n/a	n/a	n/a	n/a	n/a
$d = 5\,\mu$m	0.24	n/a	n/a	n/a	n/a
$d = 10\,\mu$m	1.81	1.52	1.27	1.02	n/a

Size estimates were calculated from the simulation results, based on Eqs. (10) and (11) (Table 1). This resulted in a consistent underestimation of size, which is expected when the timing conditions $\delta \ll \tau _D$ or $\tau _D \ll \Delta$ are violated (“n/a” entries mean that the calculation of the compartment size was not possible because of $E(q,0)=E(q,\pi )$ or lost signal modulation). Additionally, simulations varying $\tau _m$ are presented in Appendix C. Increasing mixing time results in decreasing $\cos (\psi )$ modulation, while $\cos (2\psi )$ gradually increases.

**Fig. 6:**
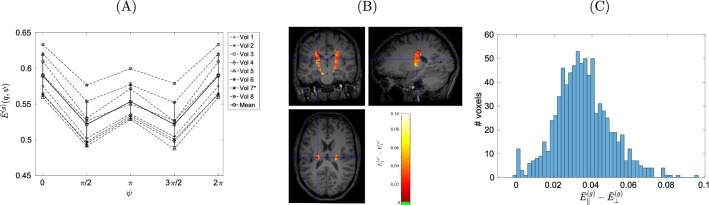
In vivo results in a ROI comprising both CST, defined using an arbitrary threshold. Voxels that did not belong to that area were manually removed. **A** Attenuation of the DDE-weighted signal vs. angle $\psi$ between the diffusion wave vectors, geometrically averaged over signals with all diffusion gradients rotated by $\pi /2$ and arithmetically averaged over the ROI. “Mean” is the arithmetic mean over all volunteers (*: $T\!E=200$ ms). The geometric mean should have removed any signal modulation due to a simple inclination with respect to the plane spanned by the diffusion gradients. The minima at $\psi =\pi /2$ and $\psi =3\pi /2$ suggest an eccentric shape of the signal-dominating compartment. **B** In vivo results for subject no. 8; parallel–perpendicular attenuation difference for the DDE-weighted signal, after taking the geometric mean over signals with all diffusion gradients rotated by $\pi /2$, in the bilateral CST ROI, overlaid with the $T_1$-weighted image. The relatively large differences found in the CST suggest the protons to reside in a more eccentric compartment, as compared to the spinal cord sample. **C** Histogram of the parallel–perpendicular differences shown in (**A**) for the ROI covering the CST

### In vivo experiments

The angular dependence of the geometric signal mean over the four perpendicular orientations of the gradient vectors in the laboratory system (i.e., over a column in Fig. 4A) can be seen in Fig. 6A. The signal profile exhibits minima at the perpendicular directions ($\psi =\pi /2$ and $\psi =3\pi /2$), which is consistent with the signal originating in an eccentric compartment. After separately averaging over parallel and antiparallel on one hand, and over the perpendicular orientations on the other [see Eq. (12)], the signal intensities from parallel orientations were significantly larger than those from the perpendicular orientations, yielding positive differences ($\bar{E}^{(g)}(q,0)-\bar{E}^{(g)}(q,\pi /2)>0$) in the ROI covering both CSTs in all volunteers. Figure 6B shows a color map of this difference in the area covering the CST in volunteer no. 8. Figure 6C shows the histogram of parallel–perpendicular differences in the CST ROI for volunteer no. 8. The SNR of the in vivo experiment was 13.04 before signal averaging (volunteer no. 8).

**Fig. 7:**
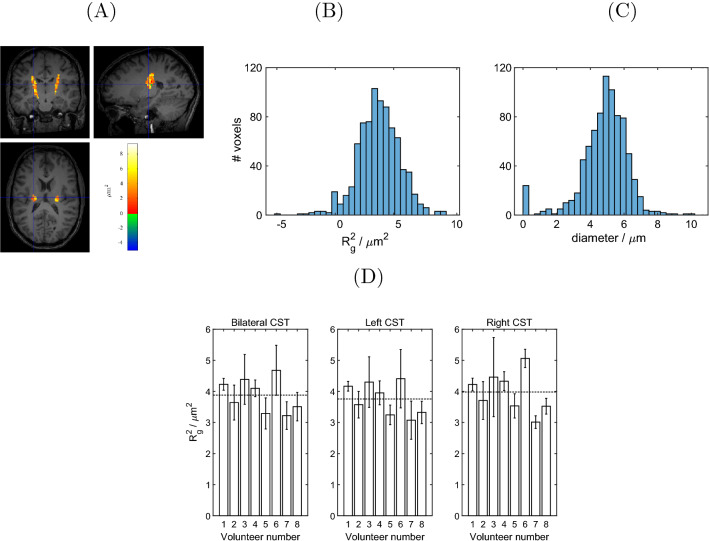
In vivo results for the size estimate. **A** size estimate $R_\text {g}^2$ [Eq. (10)] for subject no. 8, overlaid with the $T_1$-weighted image. Negative values can occur due to noise. **B** and **C** Histograms of $R_\text {g}^2$ shown in (A) and the calculated cylinder diameter [Eq. (11)] for the same volunteer. **D** Size estimate $R_\text {g}^2$ (mean over ROI) for all volunteers. The dashed lines mark the mean over volunteers. The error bars represent the standard deviation within the ROI. The $R_\text {g}^2$ mean over volunteers (± standard deviation) is $(3.9\pm 0.5)~\mu \text {m}^2$, $(3.8\pm 0.5)~\mu \text {m}^2$, and $(4.0\pm 0.7)~\mu \text {m}^2$ for the ROIs covering the bilateral, left, and right CSTs, respectively. These values correspond to estimated cylinder diameters, $2r_\text {lim}$, of $(4.6 \pm 0.3)~\mu$m, $(4.5 \pm 0.3)~\mu$m, and $(4.6 \pm 0.4)~\mu$m, respectively, according to Eq. (11)

Figure 7 shows the $R_\text {g}^2$ map (subfigure A) and the corresponding histogram (subfigure B) for volunteer no. 8. A between-volunteer comparison of the $R_\text {g}^2$ ROI mean results is displayed in Fig. 7C. The volunteer-mean of the size estimate $R_\text {g}^2$ (as shown in Fig. 7A, B for volunteer no. 8) derived from the parallel–antiparallel signal difference (Eq. (10)) in vivo was $(3.9 \pm 0.5)~\mu \text {m}$, in an area covering both CSTs (ROI mean, averaged over all volunteers). (acquired with $\tau _{\text {m}}=10.9$ ms) is in the same range as previously published results for the mean squared radius of gyration (not accounting for finite timing parameters) at $\tau _{\text {m}}=8$ ms [28]. It corresponds to a cylinder diameter of $2r_\text {lim}=(4.6 \pm 0.3)~\mu$m. Both experiments, here and in Koch and Finsterbusch [28], performed in vivo size estimations in the CST using a variation of Eq. (9). In reference [28], the arithmetic (rather than geometric) mean was used to average measurements with all gradients rotated by an integer multiple of $\pi /2$, aiming at the suppression of background gradient effects. However, the effects of the two means are very similar [28]. Note that, unlike the arithmetic mean, the geometric mean described above (as in Eq. 5) suppresses the eccentricity effect arising from tilted cylinders (see Appendix A). A statistically significant difference in $R_\text {g}^2$ between left and right CST was expected and was found for seven volunteers. Only volunteer no. 5 did not exhibit a significant left–right difference. The experiment on volunteer no. 7 on a comparison between short and long $\tau _\text {m}$ values was designed to investigate how both the parallel–antiparallel and the parallel–perpendicular attenuation differences depend on $\tau _{\text {m}}$. The statistical tests yielded that, upon increasing $\tau _{\text {m}}$, the parallel–perpendicular attenuation difference did not change significantly while the parallel–antiparallel attenuation difference decreased significantly. The latter result means that the rough compartment size estimate in Eq. (10) will underestimate the true size even more at long than at short $\tau _{\text {m}}$ values. Both test results are in accordance with expectation. (The data is shown in Appendix B).

## Discussion

### Simulations

The simulations present in this work were performed using MISST [48–51]. It allows us the calculation of the diffusion signal for arbitrary gradient waveforms for simple geometries (with known boundary conditions for the diffusion equation). However, diffusion simulation tools could provide a more realistic signal representation, such as Camino [57], 3D Realistic Microstructure Simulator (3DRMS) [58], or Disimpy [59]. In general, MC-based approaches are more computationally demanding than analytical methods like MISST. However, this issue has been solved by GPU-accelerated methods, such as in 3DRMS and Disimpy. These tools can represent complex models. 3DRMS can create substrates from microscopic images, defining binary masks. However, microscopic images conserving the integrity of the extracellular space is a problem for developing an accurate model. Lee et al. [58] proposed solutions to this issue. However, if simple geometries are assumed, a semi-analytical approach gives accurate signal calculations.

**Fig. 8:**
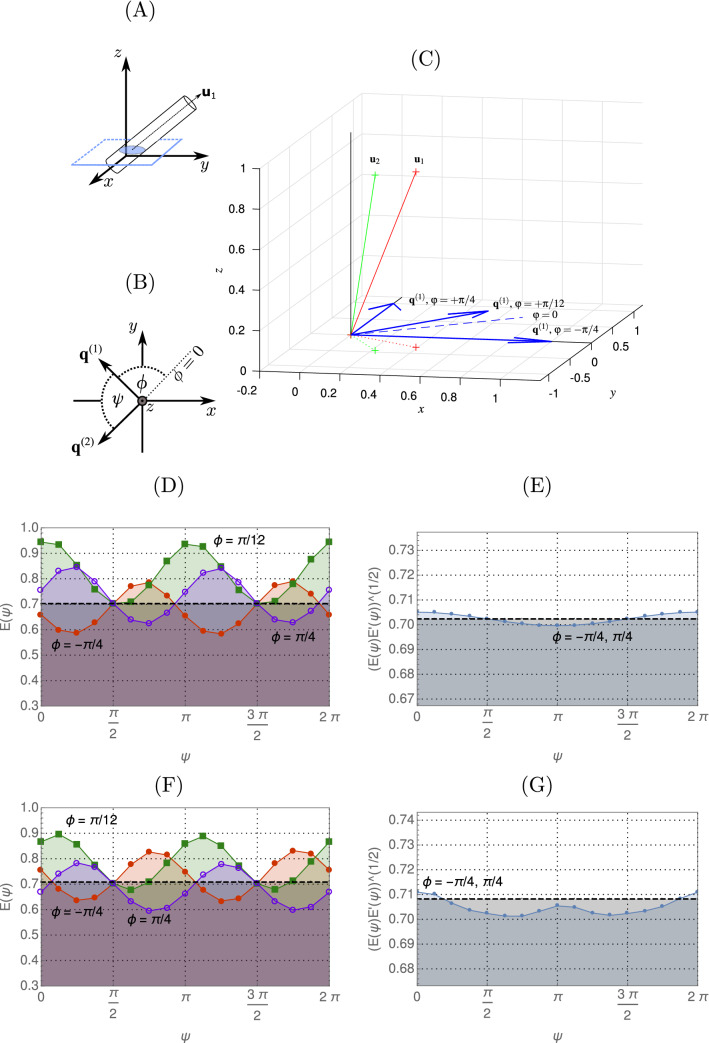
Predicted DDE signal attenuation, *E*, vs. $\psi$ (angle between the wave vectors, $\textbf{q}^{(i)}$, $i=1,2$) for circular cylinders that are inclined with respect to the *z* axis, as seen in (**A**). Diffusion gradients are in the *x*-*y* plane, as shown in (**B**) and (**C**). The plots are based on the analytic expressions from Özarslan and Basser [56] [Eq. (15)]. When varying $\psi$, one gradient is fixed while the other is rotated about the *z* axis. (The meaning of $\psi$ is as proposed in Shemesh et al. [7].) **D** Single inclined cylinder ($\textbf{u}_1$ in (C)): $E(\psi )$ for three arbitrary orientations ($\phi =-\pi /4, +\pi /12, +\pi /4$) of the non-rotating gradient, specified by the angle $\phi$ which is subtended by the fixed gradient and the direction $(x,y,z)^\textsf{T}=(1,1,0)^\textsf{T}$, (as seen in (B)). The dashed line shows $\bar{E}^{(g)}_\Vert =(\bar{E}_\Vert \bar{E}'_\Vert )^{1/2}$, i.e. the arithmetic mean of *E*(0) and $E(\pi )$, geometrically averaged over $\phi =\pm \pi /4$. **E** Geometric mean of the $E(\psi )$ curves for $\phi =-\pi /4$ and $\phi =+\pi /4$ shown in (**D**). (The geometric mean over $\phi =\phi _0$ and $\phi =\phi _0+\pi /2$ is independent of $\phi _0$.) Only effects of restriction are visible (i.e., a minimum at antiparallel orientation) while the modulation due to the cylinder inclination is removed by the geometric mean. Note the difference to Fig. 3 where the restriction effect is absent due to $\tau _{\text {m}}\rightarrow \infty$. **F, G** Signal from two cylinders with different directions ($\textbf{u}_1$ and $\textbf{u}_2$ in (**C**), see text), plotted as in (**D**) and (**E**), respectively. Subfigure (**G**) shows that in the two-fiber case the geometric means at $\psi =\pm \pi /2$ differ from $\bar{E}^{(g)}_\Vert$ (dashed line), in contrast to the single-fiber situation shown in (**E**). This was predicted on the grounds of Eq. (A5). For a single fiber orientation, this difference is always zero. (Note the different vertical scales. For more details, see text.)

When measuring how the DDE signal depends on the inter-weighting angle, a $\cos (2\psi )$ (as opposed to $\cos (\psi )$, possible at short $\tau _{\text {m}}$ only) dependence can indicate the presence of compartments that do not have rotational ($C_\infty$) symmetry in the plane of the diffusion gradients. (Strictly speaking, this dependence occurs after subtracting the mean over all $\psi$ values.) The use of a finite mixing time reduces the amplitude of the $\cos (\psi )$ modulation in a known way, resulting in underestimation of compartment size [56]. If a parallel bundle of circular cylinders is not perpendicular to the plane spanned by the diffusion gradients, the fiber cross-section is eccentric. The DDE signal then also exhibits a $\cos (2\psi )$ dependence. The main approach in this work is to use the geometric mean over perpendicular orientations of the diffusion gradient pair in the laboratory frame as a way to eliminate the signal modulation due to such a fiber inclination. This is used to investigate how far the extracellular compartment contributes to DDE-based measurements of pore size. For tilted parallel cylinders without extracellular space, Figs. 8E and 5 (case $f_i=1$) demonstrate that taking the geometric mean over the $\phi =\phi _0, \phi _0+\pi /2$ cases (with some arbitrary $\phi _0$) effectively suppresses the effect of eccentric cross-sections in the diffusion signal, resulting in a $\cos (\psi )$ profile. However, when the signal from tilted cylinders is mixed with that from an extracellular component (described by a tensor model in the simulations shown in Fig. 5), the geometric mean does not completely eliminate the signal minima at perpendicular gradient orientations. This can be attributed to diffusion along the directions perpendicular to the first tensor eigenvector (corresponding to the largest eigenvalue), which is aligned with the cylinder axis. A similar behavior is expected for a signal originating from non-parallel cylinders (see Fig. 8G).

Hence, under the assumption that the intra-axonal compartment in white matter can be modelled as cylinders with circular base, any observed parallel–perpendicular difference surviving the geometric mean, as in $\cos (2\psi )$, can be attributed to either (1) contributions from eccentric extracellular compartments that do not have a common orientation throughout the voxel or (2) fiber dispersion in the voxel. Generally speaking, in experiments using diffusion gradients in the *x*–*y* plane, both cylinders along the *z* axis but with elliptical base and inclined cylinders with circular base can induce anisotropy of the apparent diffusion coefficient in the *x*–*y* plane. The distinction between these two cases may be possible by more specialized diffusion experiments. Compartment size estimation was performed in the numerical simulations. The results showed a consistent underestimation of compartment size with increasing tensor volume fraction. This underestimation is, in general, expected when using finite experimental timing parameters. However, initially, an overestimation of pore size was expected when increasing the extracellular volume fraction. Nevertheless, as the w-modulation in our simulations arises from mixed signals of cylinder and tensor compartment, the signal modulation is gradually lost when reducing the cylinder volume fraction. The tensor approach does not provide an adequate representation of extracellular space in our case. This is because the tensor model does not have a “real” restriction due to boundaries. Hence, it is not really appropriate for studying the extracellular space influence on the DDE-based size estimate. Other approaches should be investigated, such as 3DRMS-based simulations with irregularly-shaped interconnected open pores representing the extra-axonal compartment. In Appendix D, simulations are presented that use ideal sequence parameters and a large cylinder representing the extracellular space. Compartment size overestimation can be observed in all compartments with extra-axonal volume fraction. However, as there is no eccentric compartment, a w-shaped signal modulation is not expected after taking the geometric mean.

Additionally, simulations were performed at varying mixing times (Appendix C). The impact of increasing $\tau _{\text {m}}$ can be seen there. At short $\tau _{\text {m}}$, the effects of compartmental size and shape produce a combined $\cos (\psi )\cos (2\psi )$ modulation. Upon increasing the mixing time, the $\cos (\psi )$ modulation associated with pore size vanishes slowly. A $\cos (2\psi )$ modulation persists if the compartment is eccentric. Using an intermediate mixing time, as in our simulations and in vivo experiments, allowed us to avoid overlapping gradient pulses and still obtain a signal that is affected by both modulations. This provides information on both compartment size and shape. Checking for compartment eccentricity helps to identify the origin of the diffusion signal. If the parallel–perpendicular signal difference is positive, this can mean that the signal arises not from the intra-axonal space but rather from an irregularly shaped compartment such as the extra-axonal space.

### In vivo experiments

The in vivo results shown in Fig. 6A exhibit minima at perpendicular gradient orientations, in accordance with expectation. The geometrically averaged in vivo results did show a statistically significant difference between parallel and perpendicular diffusion gradient orientations in all volunteers, including the long $\tau _{\text {m}}$ experiment performed in volunteer no. 7. Hence, the geometric mean does not successfully suppress the signal difference between parallel and perpendicular diffusion gradient orientations. This suggests the contribution of an unaligned compartment without rotational symmetry, or the presence of different fiber directions per voxel. Some degree of fiber dispersion can be found in different white matter tracts [60–62]. Indeed, the region investigated here is part of the *Corona radiata*, where the ascending CST fibers diverge. However, within the *Capsula interna*, fibers are expected to be most densely packed. Although it appears unlikely that the observed minima at perpendicular orientations purely arise from intravoxel fanning of fibers, we cannot exclude this possibility. The effects of fiber dispersion would need to be considered in future research, involving the estimation of axonal orientation distribution functions in high spatial resolution data.

This study aims at investigating whether the extracellular space contributes significantly to pore size estimates in the CST based on the parallel–antiparallel DDE signal difference [8]. Such a contribution would require that diffusion in the extracellular space exhibits signs of restriction. Interestingly, several experimental and simulation studies [63–65] concordantly concluded that the extracellular space in human brain white matter can be treated as approximately Gaussian. To what extent diffusion in a given type of tissue can be approximated as Gaussian strongly depends on the experimental conditions. One hint pointing to non-Gaussian extracellular diffusion is given by the observation that Wallerian degeneration of axons seems to reduce the restriction effect in DDE experiments [32]. Moreover, in DDE experiments on clinical hardware, it was shown that water diffusing between packed acrylate beads of 40 $\mu$m diameter exhibits restriction effects [19]. This cannot be explained by Gaussian diffusion. Although such a phantom is far from being similar to brain tissue, diffusion is likely to be hindered less there than between densely packed axons (provided no exchange occurs between intra- and extracellular spaces). Whether this notion is true or not may be subject to debate. However, the experiments described here are not based on any assumption on whether diffusion in the extracellular compartment of the CST is Gaussian or not. Unfortunately, they do not provide a firm conclusion on the contribution of the extracellular space. A confirmed contribution of the extracellular compartment would suggest that the notion of Gaussian diffusion in the extracellular space [63–65] could not be transferred to the context of these experiments. The reasons why this occurs would then need to be investigated.

In one volunteer, an additional experiment was performed that aimed at a comparison between the mixing times $\tau _\text {m,1}=10.9$ ms and $\tau _\text {m.2}=25.9$ ms. The difference between parallel and antiparallel orientations is expected to vanish at long $\tau _{\text {m}}$ [8]. We observed that the parallel–antiparallel attenuation difference is significantly higher for short than for long $\tau _{\text {m}}$ (after the geometric mean), according to expectations. The calculated size estimates $R_\text {g}^2$ observed in the CST were similar to the values found in a previous study [28], where fitting an analytic signal expression [56] to the experimental data yielded cylinder diameters of approximately $13~\mu$m. In histological studies of the human CST, axon diameters between 0.5 $\mu$m [66] and 20 $\mu$m [67] were found after fixation, where 84 % of the fibers are smaller than 2 $\mu$m [68]. In fixed human spinal cord white matter, the diameter distribution was reported to peak between 2 and 4 $\mu$m [43]. Given these values, the results presented here are in the correct order of magnitude but relatively large. A possible reason for the parallel–perpendicular difference surviving the geometric mean could be that the axonal cross-section is intrinsically eccentric, i.e. that the axonal compartment resembles a cylinder with elliptic base. In cats, the circularity index (ratio of the shortest and the largest cross-sectional diameter) was demonstrated to range from 0.8 to 0.9, approximately, and to decrease with increasing diameter [69]. Hence, the vast majority of fibers are thin and have an almost circular perpendicular cross section. However, large-diameter fibers comprise a relatively high volume and hence make a high relative contribution to results based on diffusion-weighted MR techniques. This is consistent with the tail-weighting of the size distribution as described by Veraart et al. [31]. They determined that the effective axonal radius in Corpus callosum is significantly larger in MR measurements than in histology. In other words, this overestimation suggests that larger compartments dominate the MR diffusion signal attenuation. Also, as described by Veraart et al. [31], the diffusion-weighted signal attenuation is proportional to the fourth power of the radius of a compartment, which is very weak for small pores. Therefore, low sensitivity for the intracellular compartment of thin axons might also be expected in DDE experiments, especially with weak gradient systems. This also means that the degree of overestimation arising from any possible contribution of the extracellular compartment will be difficult to estimate quantitatively.

Still, the method chosen here is not very sensitive to slight deviations from the circular shape. Overall, it appears unlikely that the intra-axonal compartment shape deviates sufficiently from a circular cylinder to induce the observed behavior. It should be noted that only few axons can be expected to have diameters above the resolution limit for diffusion-based cylinder diameter estimation using standard clinical MRI systems. This limit was estimated to lie somewhere between 4 and 8 $\mu$m [70]. In addition, it was pointed out before that DDE-based measurements are biased to larger pore diameters because the amplitude of the $\psi$ modulation increases with the pore size [28].

In this article, the quantity $R_\text {g}^2$ [Eq. (10)] was used as an estimate of the pore size. However, our experimental conditions violate the $\tau _{\text {m}}=0$ condition assumed by Mitra [8]. This results in systematic errors when characterizing microstructural properties [56], such as underestimation of compartment size and microscopic anisotropy. Typically, finite values of $\delta$, $\tau _{\text {m}}$, and $\Delta$ lead to a reduction in the modulation amplitude of the signal versus $\psi$ curve. The same can be expected for pores that do not have a completely closed cross-section. This can occur in the extracellular compartment. However, the $\delta \ll \tau _D$ condition is not met for small axons in particular. This effect is expected to increase the bias towards large axons discussed above. The total result of these combined effects is difficult to predict. The cylinder diameter itself could be derived from fitting the data with the analytic expressions describing the DDE signal [56]. In contrast to using the quantity $2r_\text {lim}$ [Eq. (11)], this would also account for finite values of $\delta$, $\Delta$, and $\tau _\text {m}$ [28]. For the purpose of the present work, however, this was not required. In part, this is because the tissue assumption of axons as parallel cylinders may be not accurate in the given situation in the CST, resulting in a rough size estimate anyway. For the given research question of whether the extracellular compartment contributes considerably, it is sufficient to discuss the dependence of the DDE signal on the interweighting angle, $\psi$. This dependence is expected to be sensitive to the shape of the compartment the signal originates in. A quantitative discussion of the size estimates derived was not intended. As discussed above, a quantitative assessment of the derived estimates and the relative compartment contributions is complicated.

*Post-mortem* studies have described anatomical differences between left and right CST axonal size [71]. In this work, a statistically significant difference in $R_\text {g}^2$ was found between left and right CST in seven out of eight volunteers. This directly translates to a difference in the estimated diameter. Here, the right CST was found to have larger compartments than the left one, where the estimated compartment size is in the same order of magnitude as in Kamiya et al. [72]. Similar left–right differences were reported previously [73]. This asymmetry is essential for the understanding of several brain illnesses as, for example, multiple sclerosis or stroke, where the asymmetry between tracts is expected to be larger than in healthy subjects [74].

A disadvantage of our theoretical approach is that it considered an over-simplistic two-compartment model assumption that can lead to wrong conclusions [75]. Since diffusion in biological tissue is in general non-Gaussian [76] and so is any restricted diffusion, a diffusion kurtosis imaging-based approach, such as correlation tensor imaging (CTI) [77] would be more appropriate. CTI is based on the cumulant expansion of the DDE signal, presented by Jespersen et al. [40]. Using the correlation tensor approach [40, 77, 78], it is possible to obtain information regarding different kurtosis sources in a biological tissue sample. This might provide a way to distinguish between extracellular space contributions and effects of orientation dispersion. However, in order to express the DDE signal in terms of the correlation tensor, it would be required to adapt the theoretical approach to the experimental conditions presented here (from a 3D to a 2D approach), which is beyond the scope of this work.

In this study, $\tau _{\text {m}}=\delta =10.9$ ms, where there is still a considerable parallel–antiparallel difference which allows a rough size estimation. Also, in volunteer no. 7, a longer $\tau _{\text {m}} = 25.9$ ms was used in order to study if different mixing times are associated with different DDE signal modulation. No statistical difference was found between parallel–perpendicular attenuation differences (after the geometric mean) for short vs. long $\tau _{\text {m}}$. In contrast, the geometrically averaged parallel–antiparallel difference decreased significantly upon increasing $\tau _{\text {m}}$. Both results are in accordance with expectation [54].

Additionally, one needs to consider that DDE experiments are also sensitive to molecular exchange between intra- and extracellular space [15, 16]. In DDE-based experiments, molecular exchange occurs at long $\tau _{\text {m}}$ and/or long $\Delta$. As the water exchange rate in healthy brain tissue is in the range between 0.4 and 1.1 $\hbox {s}^{-1}$ (according to apparent exchange rate measurements) [79]. Exchange rates around 0.8 $\hbox {s}^{-1}$ were found in the *internal capsule*, which is associated with highly myelinated axons. However, higher exchange rate values are expected in cases of myelination deficiencies and for unmyelinated axons, which corresponds to about 30 % of the fibers in the CST [80]. This is a slow time scale process which requires long $\tau _{\text {m}}$ values to detect the exchange process. Here, as our mixing time and $\Delta$ are short, it could be assumed that our experiments are not in a range in which the effects of exchange are significant.

Another concern in DDE measurements is the presence of background gradient cross-terms (BGC). These effects are known to induce DDE signal modulation in free diffusion samples at high magnetic fields [81]. However, experiments on clinical MR systems employing background gradient suppression [19] and in vivo experiments without such a suppression [28] yielded the same general signal behavior. One simple method for BGC reduction is based on the geometric mean of DWI acquisitions with all diffusion gradients inverted [82], which is associated with doubling the acquisition time. In previous comparable experiments, no signs for significant BGC were found using this approach [28].

## Conclusion

This study aimed at determining whether a pore size estimate in the CST based on double diffusion encoding measurements may rather reflect the extracellular space than the intra-axonal compartment. To this aim, it was exploited that the twice diffusion-weighted signal used for size assessment also depends on the compartment shape. This dependence leads to a signal difference between parallel and perpendicular diffusion gradient orientation. However, such a difference also occurs for cylinders with a perfectly circular base if the cylinder axis is not perpendicular to the plane spanned by the diffusion gradients. To average out the effect of such an inclination, a geometric mean was employed, taken over measurements differing by a 90$^\circ$ rotation of all diffusion gradients about the axis perpendicular to the diffusion gradients.

Eight volunteers were investigated in vivo. In all of these, a parallel–perpendicular signal difference after the geometric mean was observed. The results seem to suggest a non-negligible contribution of the interstitial compartment to the pore size estimate obtained with this double diffusion-encoding approach. However, the results are also consistent with the intra-axonal compartment being the predominant signal origin combined with a sufficiently broad orientation distribution of the axonal fibers in the voxels investigated. To rule out this alternative explanation, further experiments would be required. It should further be noted that the results presented cannot easily be transferred to experiments employing different experimental parameters as these possibly are associated with different relative signal contributions.

Care needs to be taken in analyzing size estimates calculated from DDE measurements. Mis-estimation of fiber dimensions might influence the correct diagnosis of neurological pathologies. Even though DWI is a relevant clinical tool, there is still no consensus on the origin of the changes in ADC due to, e.g., stroke [83, 84]. Determining the origin of the diffusion-weighted signal is a fundamental step for understanding the biophysical phenomena underlying pathological processes. The sensitivity of the twice diffusion-weighted signal to both pore shape and size may turn out useful for investigating subtle pathological and physiological changes in the microscopic structure of brain or other tissues. Possible applications may be possible in a number of tissues and pathologies, including pathological changes of the tissue microstructure in the corticospinal tracts. Such changes appear for instance due to the death of individual axons in motor neuron disease [85] or in Wallerian degeneration due to stroke [86].

**Supplementary information**

Extended theory is available in Appendix A.

Results regarding short and long $\tau _{\text {m}}$ experiments performed on volunteer 7 are presented in Appendix B.

Simulations with increasing $\tau _{\text {m}}$ are presented in Appendix C.

**Fig. 9:**
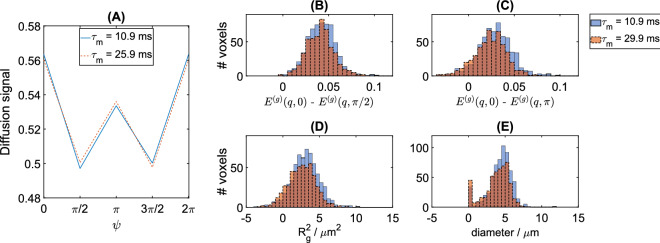
Experiments with short (10.9 ms) and long (29.9 ms) $\tau _{\text {m}}$ performed in volunteer no. 7. **A** The diffusion signal shows a W-shaped modulation for both $\tau _{\text {m}}$ values. **B** The parallel–perpendicular signal difference after the geometric mean does not exhibit a significant difference between short and long $\tau _{\text {m}}$ (Wilcoxon signed-rank test). **C** However, the parallel–antiparallel signal difference showed a statistically significant difference between long and short $\tau _{\text {m}}$, where attenuation differences for long $\tau _{\text {m}}$ were slightly smaller than for short mixing time (Mann Whitney U test). **D** shows the mean-squared radius of gyration histogram, with values of $(3.22 \pm 0.44)~\mu \text {m}^2$ and $(2.6 \pm 0.31)~\mu \text {m}^2$ for short and long $\tau _{\text {m}}$, respectively, using Eq. (10). **E** Histogram of estimated diameters calculated as in Eq. (11), resulting in $(4.39 \pm 0.23)~\mu \text {m}$ and $(3.84 \pm 0.28)~\mu \text {m}$ for short and long $\tau _{\text {m}}$, respectively

**Fig. 10:**
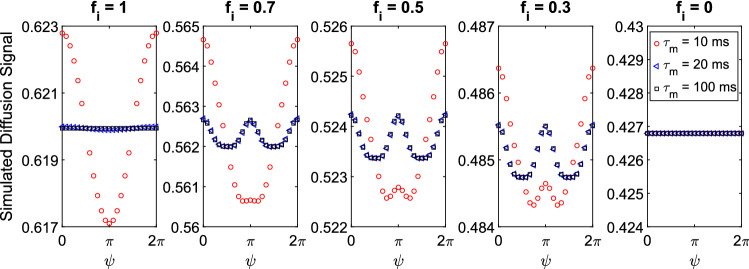
Simulated diffusion signal of different volume fractions after taking the geometric mean over $\phi = 0$ and $\phi = \pi /2$ for a cylinder and an extracellular compartment as in Fig. 5, with diameter $d = 10~\mu$m. Here, different mixing times are shown ($\tau _{\text {m}} =$ 10, 20, and 100 ms). For each $f_i$, a superposition of a $\cos (\psi )$ and a $\cos (2\psi )$ modulation is observed. Upon increasing $\tau _{\text {m}}$, the $\cos (\psi )$ modulation gradually decreases. Left to right: without the tensor compartment ($f_i=1$), the minima at $\psi =\pm \pi /2$ completely suppressed by the geometric mean mechanism. As the relative contribution of the tensor compartment increases, the minima at $\psi =\pm \pi /2$ reappear because the geometric mean mechanism is less and less efficient. At $f_i=0$, the modulation is lost completely since there is no microscopic anisotropy arising from a cylinder compartment. (Note the different vertical scales.)

**Fig. 11:**
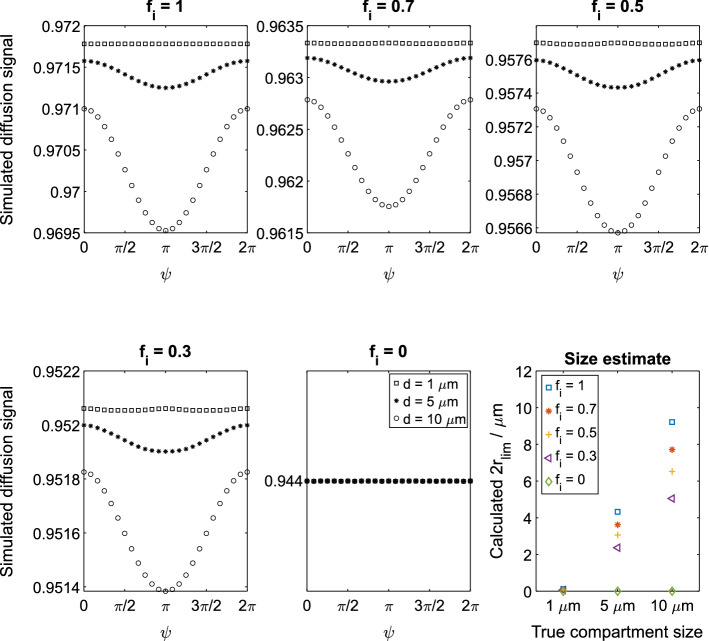
Simulations considering cylinders and a tensor describing the extracellular space, as in Fig. 5 (simulated diffusion signal of different volume fractions after the geometric mean). $f_i=1$: only cylinder (intracellular space), $f_i=0$: only tensor (extra-axonal component), and size estimate. (Note the different vertical scales.) Ideal sequence parameters were used. The w-shaped modulation is only present for small compartment sizes. The size estimate approximately corresponds to the true size for $f_i$ = 1. However, there is still a size underestimation when increasing the extra-axonal volume fraction. This is consistent with the simulations shown in Fig. 5. The tensor compartment in MISST only allows for reduced effective diffusivities but does not provide a real restriction. Therefore, under these conditions, a tensor model (in MISST) is not a good representation for the extra-axonal space. Also, one must consider that the quasi-free diffusion of the tensor reduces the modulation amplitude, resulting in size underestimation

**Fig. 12:**
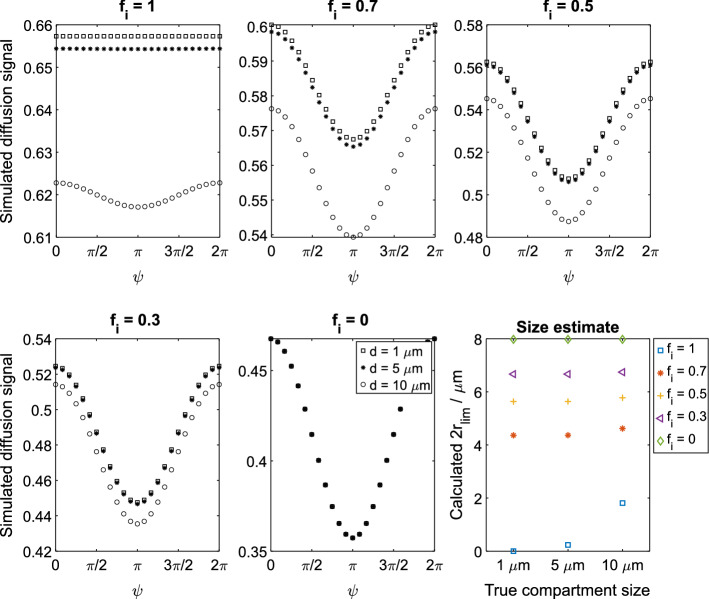
Simulated diffusion signal of different volume fractions after the geometric mean using the in vivo sequence parameters. The extracellular space is represented by a cylinder with a larger diameter (d = 20 $\mu$m, arbitrarily chosen) parallel to the other cylinders. $f_i$ = 1 (only cylinder, in three different diameters, representing intra-axonal space), $f_i$ = 0 (only large cylinder). (Note the different vertical scales.) In this situation, a w-shaped modulation is not expected. The geometric mean removes the compartment eccentricity which is due to cylinder tilting. As our in vivo sequence parameters violate Mitra’s ideal conditions ($\delta \ll \tau _D$ and $\Delta \gg \tau _D$), the compartment size is underestimated. However, this underestimation is not as severe as when using a tensor for representing extra-axonal space

**Fig. 13:**
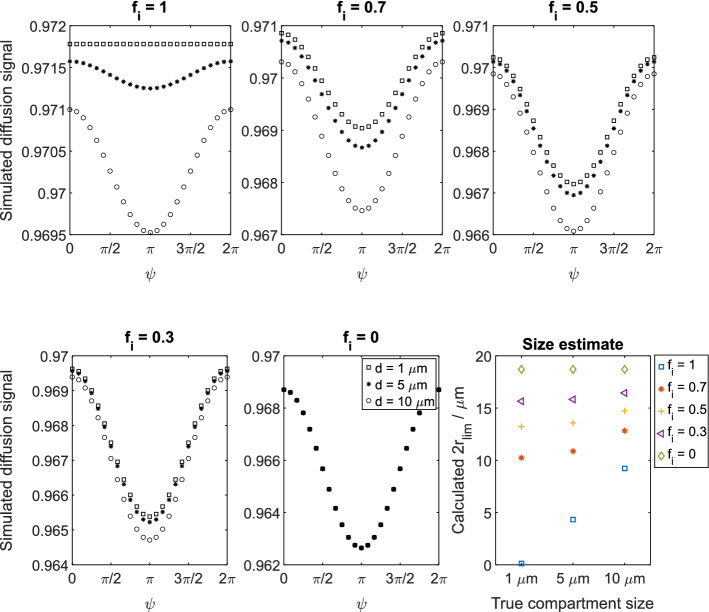
Simulated diffusion signal of different volume fractions after the geometric mean using ideal sequence parameters. The extracellular space is represented by a cylinder with a larger diameter (d = 20 $\mu$m) parallel to the other cylinders. $f_i$ = 1 (only cylinder, in three different diameters, representing intra-axonal space), $f_i$ = 0 (only large cylinder). (Note the different vertical scales.) Using ideal sequence parameters, a compartment size overestimation is present for all compartments with extra-axonal volume fraction. In this situation, a w-shaped modulation is not expected because the large tilted cylinder is parallel to the other (smaller) cylinder. The geometric mean removes the compartment eccentricity which is due to cylinder tilting

Simulations using ideal sequence parameters are presented in Appendix D. See Figs. 8, 9, 10, 11, 12, 13.

## Data Availability

Data are available upon request.

## Appendix A: Extended theory

The simple reasoning in the Theory section needs to be modified if pores of different shapes are present. Let us assume the sample consists of pores of two different shapes, with respective signal contributions of $S_a(q,\psi )$ and $S_b(q,\psi )$, for some arbitrary orientation of the diffusion gradients with respect to the sample (i.e., choice of $\phi$). The *a*-type pores are assumed to be spherical, or have $C_\infty$ symmetry with respect to an axis perpendicular to the plane spanned by the diffusion gradients, while the *b*-type pores exhibit some eccentricity in that plane with an arbitrary but identical orientation. The voxel comprises a mixture of the cases shown in the first two rows of Fig. 3. This situation could occur, for instance, in a white matter voxel containing loosely packed parallel axons which are not perpendicular to the plane spanned by the diffusion gradients. Then, the extracellular compartment would be associated with hindered diffusion of low anisotropy. After rotating all diffusion gradients by $\pi /2$ in the laboratory frame, the signal contributions may be denoted by $S'_a$ and $S'_b$. The same notation is used for the signal attenuations, *E*. Let us consider the geometric mean of the attenuations in the original and the rotated experiments,

A1 $$\begin{aligned} \sqrt{EE'}= \frac{\sqrt{(S_a+S_b)(S'_a+S'_b)}}{S_{a0}+S_{b0}}, \end{aligned}$$

where $S_{a0}$ and $S_{b0}$ represent the signal contributions when no diffusion weighting is applied. The parallel–perpendicular attenuation difference of the geometric mean is given by

A2 $$\begin{aligned} \begin{aligned}&\sqrt{E_\Vert E'_\Vert }-\sqrt{E_\perp E'_\perp } &= \left.\frac{ \sqrt{S_a^2 +S_a(S_b+S'_b)+S_bS'_b} }{S_{a0}+S_{b0}} \right|_{\psi =0} \\&\quad - \left.\frac{ \sqrt{S_a^2 +S_a(S_b+S'_b)+S_bS'_b} }{S_{a0}+S_{b0}} \right|_{\psi =\pi /2} \end{aligned} \end{aligned}$$

since $S_a=S'_a$ due to the symmetry of the *a*-type pores. For the sake of brevity, we use the alternative notation $S|_{\psi =\psi _0}:=S(q,\psi _0)$. For $\tau _\text {m}\rightarrow \infty$, $S_a=S'_a$ are independent of $\psi$ [8]. At finite values of $\tau _\text {m}$ or zero, however, this is not true: even the spherical pore signal $S_a$ has a cosine dependence on $\psi$ [56]. It is possible that in some experiments $\tau _\text {m}$ is not large enough to make the size effect (i.e. the additional cosine dependence of $S(q,\psi )$) vanish. A cosine dependence would also induce $S(q,0) \ne S(q,\pi /2)$ for pores of all shapes. As we are interested in the parallel–perpendicular difference which arises for eccentric pores only, we would like to remove the additional size effect. To this aim, we replace Eq. (A2) by a simpler equation through considering MR signals that are averaged over parallel and antiparallel orientations as suggested previously [77]. We denote this average with a bar, $\bar{S}_a|_{\psi =\psi _0}=(S_a|_{\psi =\psi _0}+S_a|_{\psi =\psi _0+\pi })/2$. (It should be noted that $\overline{S_a+S_b}=\bar{S}_a+\bar{S}_b$ is directly observable without knowledge of the two individual pore type contributions). For simplicity, we first omit the square root and consider the difference between the attenuation products themselves. With $\bar{E}=\overline{(S_a+S_b)}/(S_{a0}+S_{b0})=(\bar{S}_a+\bar{S}_b)/(S_{a0}+S_{b0})$, we obtain

A3 $$\begin{aligned} \begin{aligned}&\bar{E}_\Vert \bar{E}'_\Vert -\bar{E}_\perp \bar{E}'_\perp = \frac{1}{(S_{a0}+S_{b0})^2} \\&\left( \bar{S}_a |_{\psi =0} \left[ (\bar{S}_b+\bar{S}'_b) |_{\psi =0} - (\bar{S}_b+\bar{S}'_b) |_{\psi =\pi /2} \right] \right. \\&\quad \left. + \left[ (\bar{S}_b \bar{S}'_b) |_{\psi =0} - (\bar{S}_b \bar{S}'_b) |_{\psi =\pi /2} \right] \right) . \end{aligned} \end{aligned}$$

It was used here that $\bar{S}_a|_{\psi =0}=\bar{S}_a|_{\psi =\pi /2}$. This is a consequence of the cosine dependence of $S_a$, i.e. $E=S_a/S_{a0}=1-K+L\cos (\psi )$ with some real constants *K* and *L* [56], and $\cos (\psi )=-\cos (\psi +\pi )$, meaning that $\bar{S}_a$ is independent of $\psi$. Recalling that the term in the second pair of square brackets is zero, as argued above when deriving Eq. (5), leads to

A4 $$\begin{aligned} \bar{E}_\Vert \bar{E}'_\Vert -\bar{E}_\perp \bar{E}'_\perp= & {} \frac{1}{(S_{a0}+S_{b0})^2} \bar{S}_a |_{\psi =0} \left[ (\bar{S}_b+\bar{S}'_b) |_{\psi =0}\right. \nonumber \\{} & {} \left. - (\bar{S}_b+\bar{S}'_b) |_{\psi =\pi /2} \right] , \end{aligned}$$

which will vanish for $\bar{S}_b\equiv 0$ but also for $\bar{S}_a\equiv 0$. If, however, neither $\bar{S}_a$ nor $\bar{S}_b$ are zero, the term in the square brackets in Eq. (A4) is also not zero, for the same reason as argued for Eq. (3). Again, this can be seen by assuming an exponential decay with effective diffusion coefficients and expanding $\bar{S}_b$ and $\bar{S}'_b$ to second order in *b* (i.e., to 4th order in *q*).

We need to consider cases with different volume fractions for “a” and “b” type pores. For this purpose, we define $0 \le f_a\le 1$ as the volume fraction of the non-eccentric “*a*” pores and set $\bar{S}_a=f_a\bar{s}_a$, $\bar{S}_b=(1-f_a)\bar{s}_b$, and $\bar{S}'_b=(1-f_a)\bar{s}'_b$. The signal which would arise from a voxel exclusively containing “a” or “b” type pores is given by $s_a$ and $s_b$, respectively. The meaning of the overbar and prime notations is unchanged. Eq. (A4) can then be rewritten to

A5 $$\begin{aligned} \bar{E}_\Vert \bar{E}'_\Vert -\bar{E}_\perp \bar{E}'_\perp= & {} \frac{1}{(S_{a0}+S_{b0})^2} f_a\nonumber \\{} & {} (1-f_a) \bar{s}_a |_{\psi =0} \left[ (\bar{s}_b+\bar{s}'_b) |_{\psi =0} - (\bar{s}_b+\bar{s}'_b) |_{\psi =\pi /2} \right] , \end{aligned}$$

which tends to zero as $f_a\rightarrow 0$ or $f_a\rightarrow 1$. In fact, $f_a(1-f_a)=-(f_a-1/2)^2+1/4$ attains a maximum at $f_a=1/2$. The term $S_{a0}+S_{b0}$ is independent of $f_a$. In summary, this means that for the given geometry, the difference in Eq. (A5) does not vanish if significant contributions of both non-eccentric and aligned eccentric pores exist.

Figure 8 shows the dependence of the signal attenuation on $\psi$ for two geometrical situations: an inclined cylinder (D, E) and a combination of two cylinders inclined in different directions (F, G). The diffusion gradients are assumed to be in the *x*–*y* plane, as illustrated in Fig. 8B, C. The plots were calculated using the analytic expressions for given exact experimental parameters provided by Özarslan and Basser [56] (Eq. (15), accounting for the $\pi$ shift arising from $\psi$ being the angle subtended by the $\textbf{q}^{(i)}$ vectors in the present paper rather than the gradient vectors as in [56].) for inclined circular cylinders and finite values of $\delta , \Delta , \tau _\text {m}$, using Mathematica 11.3 (Wolfram, Champaign, Illinois), terminating the infinite series as in [28]. Three different arbitrary orientations of the non-rotating diffusion gradient with respect to the cylinders are shown, as depicted in 8 C). It can be seen that for the example comprising two cylinders inclined in different directions, $\bar{E}^{(g)}_\Vert$ does not coincide with $(E(q,\psi )E'(q,\psi ))^{1/2}$ at $\left| \psi \right| = \pi /2$. (Parameters used for the calculation: $G=80$ mT $\hbox {m}^{-1}$, $\tau _\text {m}=5.9$ ms, $\delta =5$ ms, $\Delta =50$ ms, cylinder diameter $a=4~\mu$m, diffusion coefficient [87] $D_0=2.25\cdot 10^{-9}~\text {mm}^2\,\text {s}^{-1}$. For Fig. 8D and E, the cylinder orientation was set to $\textbf{u}_1=\textsf{R}_z(2\pi /7)\textsf{R}_x(\pi /6)(0, 0, 1)^\textsf{T}$, where $\textsf{R}_x(\alpha )$ and $\textsf{R}_z(\alpha )$ correspond to a rotation about the *x* and *z* axis, respectively. For Fig. 8F and G, a second cylinder with $\textbf{u}_2=\textsf{R}_z(-\pi /7)\textbf{u}_1$ was added($\angle (\textbf{u}_1, \textbf{u}_2)\approx 12.8^\circ$) and the $E(\psi )$ values for the two cylinders were averaged.)

## Appendix B: Comparison short vs. long mixing time in vol. no. 7

See Fig. 9.

## Appendix C: Simulations varying the mixing time

See Fig. 10.

## Appendix D: Simulations with ideal conditions

Here, we aim to explain the size underestimation shown in Table 1. As mentioned in the discussion, there are several factors influencing these results. In this appendix, simulations with ideal and in vivo sequence parameters were performed. These parameters were determined using the definition for the small *q* regime by Özarlan and Basser [56] ((2$\pi$qa)$^2 \ll 1$, where $q = \delta G \gamma$, and *a* corresponds to the compartment size), and $\delta \ll \tau _D$ (note that $\delta \ll \tau _D$ does not hold for the diameter 1 $\mu$m). Accordingly, as “ideal parameters” the following were chosen: $\delta$ = 0.1 ms, $\Delta$ = 0.4 s, *G* = 0.44 T $\hbox {m}^{-1}$, resulting in *q* = 11770 $\hbox {m}^{-1}$. The mixing time is $\tau _{\text {m}} = \delta$.

### D.1 Simulation considering cylinders of three different diameters as intra-axonal space and a tensor as extra-axonal space

See Fig. 11.

### D.2 Simulation considering cylinders of three different diameters as intra-axonal space and cylinder with larger diameter as extra-axonal space

As mentioned before, the tensor model fails to represent compartmental restriction. Therefore, here we simulate the diffusion signal by representing the extra-axonal space by a compartment with a true restriction. A cylinder with a larger diameter is chosen to represent the extracellular space. Simulations are performed using ideal ($\delta$ = 0.1 ms, $\Delta$ = 0.4 s, *G* = 0.44 T/m, see Fig. 13) and our in vivo ($\delta$ = 10 ms, $\Delta$ = 0.062 s, *G* = 0.044 T/m, see Fig. 12) sequence parameters. The mixing time is $\tau _{\text {m}} = \delta$.

## Abbreviations

DDE: Double diffusion encoding
DWI: Diffusion weighted imaging
DTI: Diffusion tensor imaging
CST: Corticospinal tract

## Code availability

As per our research agreement with Philips Healthcare the MR code used in this study is confidential.
